# The mediating role of hope in the relationship between purpose in life and anxiety: A cross-cultural analysis in university students

**DOI:** 10.1371/journal.pone.0331042

**Published:** 2025-09-08

**Authors:** Esteban Moreno-Montero, Rodrigo Moreta-Herrera, Jose A. Rodas, Evelyn Cuesta-Andaluz, Diana Ximena Puerta-Cortés, Daniela Ferrufino-Borja, Daniel Oleas, Renzo Gismondi Diaz

**Affiliations:** 1 Escuela de Psicología, Pontificia Universidad Católica del Ecuador, Ambato, Ecuador; 2 Escuela de Psicología, Universidad Espíritu Santo, Samborondón, Ecuador; 3 School of Psychology, University College Dublin, Dublin, Ireland; 4 Facultad de Ciencias de la Salud, Universidad Internacional de La Rioja, La Rioja, Spain; 5 Programa de Psicología, Universidad de Ibagué, Ibagué, Colombia; 6 Facultad de Ciencias de la Salud, Universidad Católica del Maule, Talca, Chile; 7 Escuela de Psicología, Universidad Ecotec, Samborondón, Ecuador; 8 Facultad de Humanidades, Comunicación y Artes, Universidad Privada de Santa Cruz de la Sierra, Santa Cruz de la Sierra, Bolivia; Norbert Wiener University, PERU

## Abstract

**Objective:**

To examine the direct effect of purpose in life (PIL) on anxiety and its indirect effect through hope in a sample of university students from Ecuador, Bolivia, and Colombia, while assessing the cross-national measurement equivalence of the mediation model.

**Method:**

A descriptive, correlational, and cross-sectional study was conducted using Structural Equation Modelling (SEM) to test mediation effects and Multigroup Confirmatory Factor Analysis (MG-CFA) to assess measurement invariance. The sample included 1,459 university students from Ecuador, Colombia, and Bolivia.

**Results:**

PIL, hope, and anxiety exhibited significant latent correlations and formed a well-fitted structural model. PIL had both a direct effect on anxiety in Colombia and an indirect effect through hope across all three countries. In Ecuador and Bolivia, full mediation was observed, whereas in Colombia, mediation was partial. PIL and hope together explained 20.1% of the variance in anxiety, though this percentage varied across countries. Measurement invariance analyses confirmed that the mediation model was structurally equivalent across cultures, but differences in effect sizes suggest cultural modulation in the relationship between PIL, hope, and anxiety.

**Conclusion:**

Anxiety were associated with cognitive processes related to meaning-making and hope, supporting their role as protective psychological variables. However, cultural context modulates the strength of these relationships, highlighting the need for context-sensitive interventions.

## Introduction

Anxiety is an anticipatory emotional state triggered by situations perceived as dangerous, motivating alertness and readiness for action. While it can be part of everyday life, excessive and prolonged anxiety may develop into a mental disorder [1], manifesting in various anxiety disorders [2].

Evidence indicates a high prevalence of anxiety among university students [3], with rates ranging from 28% to 54% [4–6], which may negatively impact academic performance [7]. Therefore, monitoring this condition is essential to understanding its progression and developing intervention strategies.

In this regard, research has increasingly explored alternative approaches to promoting mental health in university students. From the perspective of Positive Psychology, aspects such as gratitude, humility, and hope contribute to psychological well-being [8]. However, these factors have received less attention in psychological research due to the predominant focus on psychopathology [9–11]. Enhancing these aspects has been suggested as a means of managing psychological symptoms [12], including those of anxiety [13], although further research is needed to explore their impact and scope.

### Purpose in life and hope

In university life, two fundamental psychological components are purpose in life (PIL) and hope. PIL, introduced by Viktor Frankl [14], is defined as an organising system that guides goals and actions. Operationally, it involves the perception of a positive purpose that drives individuals forward [15], and it is a key [16][17] of both psychological well-being and emotional regulation.

Within the university context, PIL fosters academic achievement [18], provides meaning to effort, and strengthens life purpose [19]. Its absence can lead to existential emptiness and neuroticism [20], whereas its presence promotes mental health and prevents disorders such as suicidal ideation [21–23] and depressive symptoms [24]. Regarding anxiety, there is evidence of mild to moderate negative correlations in the general population [25,26], but not among university students, representing a research gap.

Hope, or hopeful thinking, refers to the positive expectation of achieving desired outcomes. It is not merely a feeling but a cognitive-emotional and contextual structure [27]. According to authors such as Herth [28,29], hope involves: (a) cognitions and determination regarding the likelihood of future success, (b) confidence and actions that facilitate its achievement, and (c) spiritual connection and interpersonal relationships as sources of strength.

Hope has gained significance in research as a positive psychological resource. It is crucial for academic success [30,31] as it enables students to cope with challenges and graduation requirements. It also predicts well-being and positive mental health [32–34], while mitigating anxiety symptoms and anxiety disorders in the general population [35,36] as well as in university students [37,38]. Furthermore, research suggests that hope may play a mediating role in the relationship between psychological risk factors and suicidal ideation [39]. It is considered a protective factor against anxiety [40], though further exploration is needed in this field.

### The present study

As previously noted, PIL and hope are key psychological attributes in the course and development of academic life, particularly in achieving goals, as well as in the mental health of university students. These variables even covary with each other [41], with PIL acting as a predictor of hope [42]. However, research on these constructs remains limited, requiring further attention and development. The first limitation is that current studies primarily assess their covariance with anxiety independently, focusing largely on the general population and, to a lesser extent, on university students. Research has not yet extended to identifying the predictive potential of each construct or proposing a multiple explanatory model of anxiety based on these positive factors, despite their recognition as protective and resilience-enhancing elements against anxiety [26,40].

Additionally, the role of these potential predictors has not been examined in terms of the direct and indirect effects they may exert on anxiety or their participation as mediating variables. Existing evidence suggests that hope functions as a mediating variable in mental distress [43]. Furthermore, due to the scarcity of data in South American populations, the prevalence and intensity of these variables in university students remain unclear, as does the potential influence of cultural and national conditions. Considering national groups from Ecuador, Colombia, and Bolivia as reference points, it is unknown how these contextual factors might shape the strength and intensity of the interactions among the variables of interest.

In this regard, developing an explanatory model, along with a mediation model of anxiety and a cross-cultural invariance analysis through positive predictors, could enhance understanding of the phenomenon under study. This would facilitate the identification of underlying mechanisms in specific relationships [44] and support intervention strategies for managing anxiety in university populations.

Based on these considerations, the study aims to: a) Establish a model of fit between PIL, hope, and anxiety in a sample of university students from Ecuador, Bolivia, and Colombia; b) Propose a structural mediation model of anxiety derived from PIL, considering hope as a mediating variable; and c) Determine the transnational equivalence of the proposed mediation model.

From these objectives, the study hypotheses that: (H1) There is an adequate model fit for PIL, hope, and anxiety in the selected sample, (H2) PIL has a direct effect on anxiety and an indirect effect through hope as a mediating variable and (H3) The proposed mediation model demonstrates cross-cultural equivalence in the referenced South American population.

## Methods

### Participants

The study included 1,459 university students from three South American countries: Bolivia, Colombia, and Ecuador. The sampling was non-probabilistic, based on inclusion criteria and voluntary participation. The inclusion criteria were: (a) residing in one of the participating countries; (b) being enrolled in an undergraduate programme at a higher education institution in one of these countries; (c) reading and accepting the informed consent form; and (d) completing all sections of the survey.

Table 1 presents the demographic data of the participants. Some similarities were observed across countries, although differences were also present in certain categories. The average age was 20.2 years, with a higher predominance of female participants. The majority resided in urban areas. Regarding fields of study, health sciences programmes accounted for the highest percentage of students in all three countries. Additionally, most participants reported dedicating themselves exclusively to their studies without engaging in employment.

**Table 1:** Participants’ demographic characteristics.

	Bolivia (n = 474)	Colombia (n = 362)	Ecuador (n = 623)
Age (*M* ± *SD*)	20.6 ± 2.9	19.9 ± 2.9	20.0 ± 2.4
**Sex, n (%)**			
Male	165 (34.8%)	146 (40.3%)	252 (40.4%)
Female	309 (65.2%)	216 (59.7%)	371 (59.6%)
**Place of residence**			
Urban	426 (89.9%)	352 (97.2%)	503 (80.7%)
Rural	48 (10.1%)	10 (2.8%)	120 (19.3%)
**Field of study**			
Arts and Humanities	22 (4.6%)	7 (1.9%)	19 (3.1%)
Sciences	0 (0%)	5 (1.4%)	3 (0.5%)
Health Sciences	295 (62.2%)	170 (47.0%)	356 (57.1%)
Social Sciences and Law	47 (9.9%)	117 (32.3%)	197 (31.6%)
Engineering and Architecture	110 (23.2%)	63 (17.4%)	48 (7.7%)
**Employment Status**			
Studies and does not work	302 (63.7%)	241 (66.6%)	505 (81.1%)
Studies and works	172 (36.6%)	121 (33.4%)	118 (18.9%)

### Instruments

#### Purpose in Life Scale Short Form (PIL-SF).

This is a brief scale developed by Crumbaugh & Maholick [15,45] consisting of four items designed to assess the experience of purpose in life. Items are rated on a seven-point Likert scale. The total score is obtained by summing the responses, with a possible range of 4–28 points, where higher values indicate a stronger sense of PIL. In its original version, the instrument demonstrated adequate internal reliability (α = .84). Additionally, previous studies in the Ecuadorian population validated its unidimensional structure and confirmed its high internal consistency (α = .85) [9].

#### Generalised Anxiety Disorder Scale (GAD-7).

This scale was designed by Spitzer et al. [46,47] to assess anxiety symptoms commonly present in Generalised Anxiety Disorder through seven items, each rated on a four-point Likert scale ranging from 0 (“Not at all”) to 3 (“Nearly every day”). The total score is obtained by summing all items, with a possible range of 0–21 points. Furthermore, the GAD-7 retained its unidimensional structure and showed very high internal reliability (ω = .91) in the Ecuadorian population [44].

#### Herth Hope Index (HHI).

Designed by Herth [29,48] to measure hope, this index consists of 12 items rated on a four-point Likert scale, ranging from 1 (“Strongly disagree”) to 4 (“Strongly agree”). These items are grouped into three factors: (a) Temporality and Future, (b) Positive readiness and expectancy, and (c) Interconnectedness. In the original study, the instrument demonstrated excellent internal consistency (α = .97). Similarly, in an analysis conducted on Ecuadorian university students, its reliability was equally high (ω = .97) [49].

Although various factorial models have been proposed and tested for the HHI, in the present study, hope was conceptualised as a global construct, and the unidimensional model previously analysed in the Spanish population was adopted [50].

### Procedure

Data collection was conducted simultaneously across the three countries using surveys administered via Google Forms. To facilitate this process, the research team first contacted the selected educational institutions and, upon obtaining authorisation, the survey link was distributed. On the first page of the online form, participants were presented with an informed consent statement outlining the purpose of the study, the voluntary nature of participation, the right to withdraw at any time without penalty, and assurances of anonymity and confidentiality. Only individuals who indicated their agreement by selecting the corresponding option were granted access to the questionnaire, thus providing written informed consent in digital form. The estimated time to complete the survey was approximately 15 minutes and all participants were adults.

Data collection took place between February and May 2023. Subsequently, the results were organised into a digital database, inconsistent responses were filtered out, and statistical analyses were conducted in line with the study’s objectives. At all stages of the study, ethical standards outlined in the Declaration of Helsinki for research involving human participants were followed, as well as the ethical and regulatory guidelines established by the participating universities. In addition, the research protocol was approved by the Research Committee of the Psychology Department at Universidad Tecnológica Ecotec (No 05-30-01-2023).

### Data analysis

The data analysis was conducted in five phases.

In the first phase, descriptive statistics were calculated to assess the levels of the constructs by country, and univariate normality was examined (*g₁, g₂* ~ 1.5) [51]. For multivariate normality, Mardia’s test [52] was applied, where *p* > .05 indicated no significant deviations.

In the second phase, Confirmatory Factor Analyses (CFA) were performed separately for each country and instrument. Given the violation of multivariate normality and the ordinal nature of the data, the Diagonally Weighted Least Squares (DWLS) estimator was used [53]. Unidimensionality was assessed based on previous models [9,44,50]. Model fit was evaluated using indices such as Chi-square (χ^2^), normed Chi-square (χ^2^/*df*), Root Mean Square Error of Approximation (RMSEA), and Standardised Root Mean Square Residual (SRMR). A good fit was determined by RMSEA and SRMR values below.08, a non-significant Chi-square (*p* > .05), and a normed Chi-square below 5 [54,55]. Additionally, incremental fit indices such as the Comparative Fit Index (CFI) and Tucker-Lewis Index (TLI) were calculated, with values above .95 considered indicative of excellent fit [56,57].

In the third phase, latent correlations were estimated using Structural Equation Modelling (SEM), and the model fit was assessed. Structural regressions were then incorporated to analyse the mediation effect of hope between PIL and anxiety. Mediation was considered present when the indirect effect (a*b) was significant (*p* < .05). If the direct effect (*c’*) was non-significant (*p* > .05), full mediation was concluded; otherwise, partial mediation was determined [58,59]. The coefficient of determination (**R*^*2*^*) was also calculated to establish the percentage of variance explained by the model for anxiety in each country.

In the fourth phase, Measurement Equivalence (ME) was examined using Multigroup Confirmatory Factor Analysis (MG-CFA). An initial unrestricted model was established, followed by progressively constrained models to evaluate metric, scalar, and strict equivalence [60,61]. To mitigate biases related to sample size, ME was verified through ΔCFI (< 0.01) and ΔRMSEA (< 0.015) [62].

Finally, in the fifth phase, a Multigroup SEM (MG-SEM) analysis was conducted to determine whether nationality influenced direct and indirect effects. Hierarchical models were compared by imposing equality constraints on each effect. Equivalence was established when fit indices (CFI, TLI, SRMR, RMSEA) remained stable or improved compared to the baseline model, and Δχ^2^ was non-significant [63]. Additionally, the Chi-square difference test (Δχ^2^) was used, where a non-significant value indicated that the constrained model had a fit similar to the initial model, supporting the equivalence of the restricted effect [64].

All statistical analyses were conducted using the R programming language within the R-Studio virtual environment [65], employing the *psych*, *lavaan*, and *semTools* packages.

## Results

### Preliminary analysis of the measures

Table 2 presents the descriptive statistics of the variables. Regarding anxiety levels, participants from Colombia and Ecuador exhibited low levels of anxiety, whereas participants from Bolivia reported moderate levels. In terms of hope, the average scores reflected moderate levels of hope across all groups. Additionally, the mean PIL scores were high in all countries, suggesting a strong presence of purpose in life.

**Table 2:** Descriptive statistics and Mardia’s test for normality of study variables by country.

*Country*	*Factors*	*M*	*SD*	*g*_*1*_	*g*_*2*_	*Mardia’s test*
Bolivia	Purpose in life	19.99	5.07	−0.59	−0.03	*g*_*1* _= 284.94***
						*g*_*2*_ = 18.08***
	Hope	34.62	6.81	−0.40	−0.31	*g*_*1* _= 1059.65***
						*g*_*2*_ = 23.26***
	Anxiety	10.10	5.35	0.15	−0.78	*g*_*1* _= 163.47***
						*g*_*2*_ = 5.78***
Colombia	Purpose in life	21.80	4.76	−0.79	0.31	*g*_*1* _= 150.52***
						*g*_*2*_ = 5.96***
	Hope	37.07	6.67	−0.55	−0.01	*g*_*1* _= 1220.66***
						*g*_*2*_ = 23.06***
	Anxiety	8.14	5.47	0.49	−0.61	*g*_*1* _= 266.02***
						*g*_*2*_ = 10.50***
Ecuador	Purpose in life	21.24	5.29	−0.89	0.37	*g*_*1* _= 468.76***
						*g*_*2*_ = 26.78***
	Hope	35.60	7.21	−0.53	0.02	*g*_*1* _= 1376.79***
						*g*_*2*_ = 33.74***
	Anxiety	9.72	5.65	0.22	−0.88	*g*_*1* _= 220.45***
						*g*_*2*_ = 19.21***

*** = *p* <.001; *M* = Arithmetic mean; *g*_*1*_ = skewness; *g*_*2*_ = kurtosis.

In terms of distribution, the skewness (*g₁*) and kurtosis (*g₂*) indices for all variables were within the ~ 1.5 range, indicating compliance with univariate normality. However, the data did not meet the criteria for multivariate normality, as Mardia’s test was significant (*p* < .05).

### Confirmatory factor analysis of the measures

Table 3 presents the fit index values for the unidimensional models assessed in each country. Among the results, the PIL-SF showed the strongest fit indices, followed by the GAD-7 and HHI, which also demonstrated adequate fit, although the chi-square test was significant. Additionally, the factor loadings for all items exceeded the minimum acceptable threshold (λ > .40), supporting strong relationships with their respective latent constructs. No structural modifications by country were necessary, nor was the removal of any items from the instruments.

**Table 3:** Fit indices of measurement models for PIL-SF, HHI, and GAD-7 across countries.

Country	Measures	*χ* ^*2*^	*df*	*χ* ^*2*^ */ df*	CFI	TLI	SRMR	RMSEA
Bolivia	PIL-SF	0.73^+^	2	0.37	.99	.99	.007	.001 [.001;.068]
	HHI	226.25***	54	4.19	.98	.98	.072	.082 [.071;.093]
	GAD-7	12.49^+^	14	0.89	.99	.99	.022	.001 [.001;.040]
Colombia	PIL-SF	0.13^+^	2	0.07	.99	.99	.004	.001 [.001;.026]
	HHI	127.29***	54	2.36	.99	.99	.062	.061 [.048;.075]
	GAD-7	13.24^+^	14	0.95	.99	.99	.025	.036 [.001;.048]
Ecuador	PIL-SF	0.96^+^	2	0.48	.99	.99	.007	.001 [.001;.064]
	HHI	206.99***	54	3.83	.99	.99	.055	.067 [.058;.077]
	GAD-7	25.59*	14	1.83	.99	.99	.025	.036 [.011;.059]

*** = **p* *< .001; * p < .05; *+ p > *.05; *χ*^*2*^* *=* *chi-squared; df = degrees of freedom; χ^2^/gl = normed chi-squared; CFI = Comparative fit index; TLI = Tucker Lewis Index; RMSEA = Root mean square error of approximation; SRMR = Standardized Root Mean Square Error Residual.

### Structural analyses

#### Overall model fit.

Regarding the overall fit of the model among the latent variables, Table 4 presents the fit indices for each country. The results indicate that, except for the chi-square test, which was significant (*p* < .05) in all countries, the remaining fit indices were optimal. Therefore, it is confirmed that the general correlation model is suitable for representing the latent relationships between the constructs across the different countries.

**Table 4:** Overall model fit indices for the latent variable structure across countries.

Country	*χ* ^*2*^	*df*	*χ* ^*2*^ */ df*	CFI	TLI	SRMR	RMSEA
Bolivia	847.98***	227	3.74	.98	.97	.080	.076 [.071;.082]
Colombia	553.78***	227	2.44	.99	.99	.071	.063 [.056;.070]
Ecuador	1026.02***	227	4.52	.99	.99	.071	.075 [.071;.080]

*** = **p* *< .001; *χ*^*2*^* *=* *chi-squared; df = degrees of freedom; χ^2^/gl = normed chi-squared; CFI = Comparative fit index; TLI = Tucker Lewis Index; RMSEA = Root mean square error of approximation; SRMR = Standardized Root Mean Square Error Residual.

**Fig 1:**
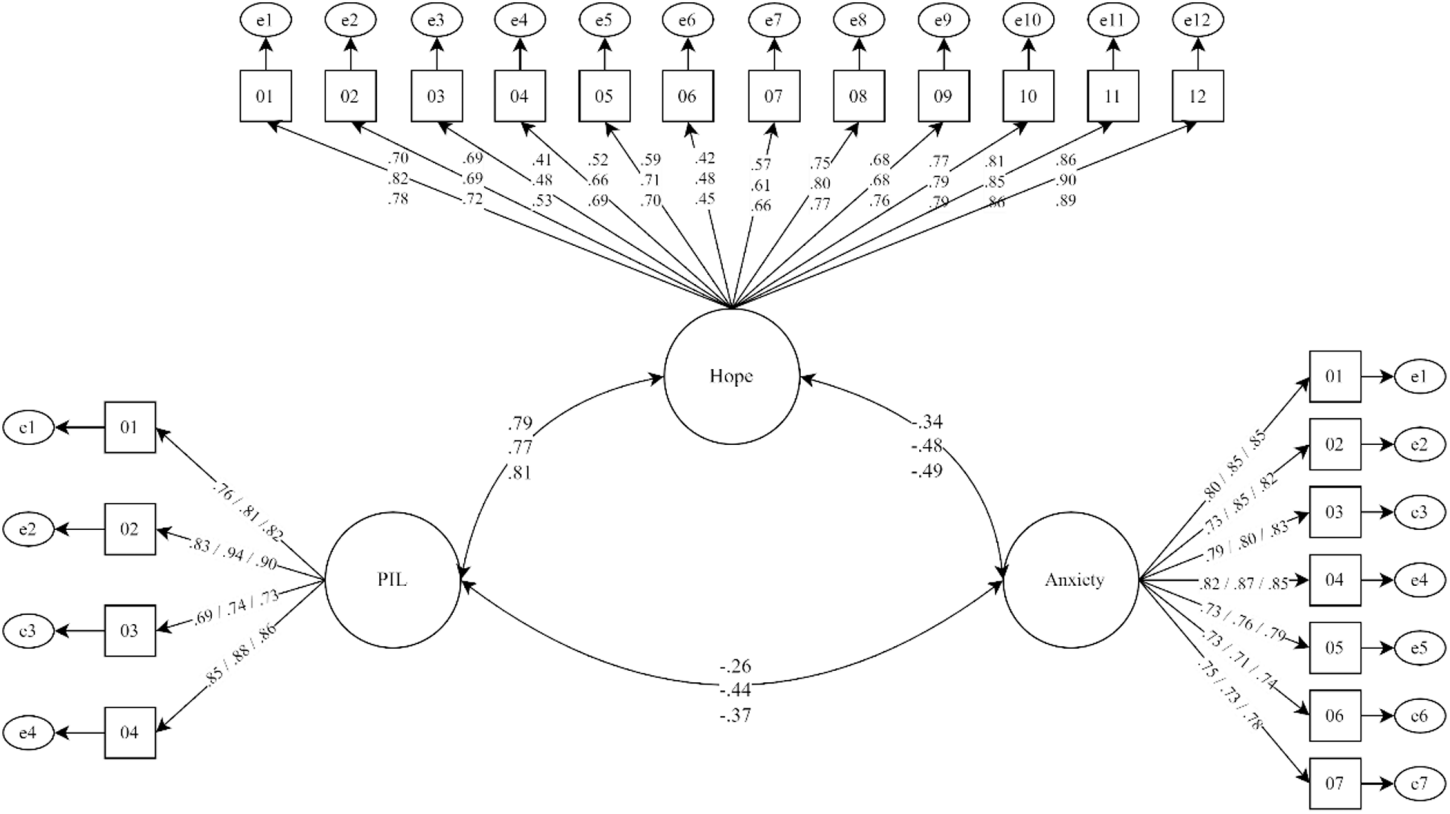
Structural equation model of the relationships between purpose in life, hope, and anxiety. χ2(831) = 2622.77; p < .05; CFI = .99; TLI = .99; RMSEA = .067 IC90% [.064 −.070]; SRMR = .074. Bidirectional lines represent latent relationships. All coefficients were significant at p < .001. Data are presented in the following order: Bolivia/ Colombia/ Ecuador.

Fig 1 presents the overall model fit, the relationships between latent factors, and the factor loadings by country. The strongest correlation observed across all countries was between PIL and hope, with the highest value recorded in Ecuador, indicating a strong association between these variables. On the other hand, hope showed a moderate negative correlation with anxiety in each country, with the strongest effect also observed in Ecuador. The inverse relationships between anxiety and the HHI were larger than the negative correlations between PIL and anxiety, with the highest correlation found in Colombia. Additionally, all items had factor loadings above the recommended threshold (λ > .40), confirming their significant contribution to their respective constructs and the overall model.

### Structural mediation analysis

**Fig 2:**
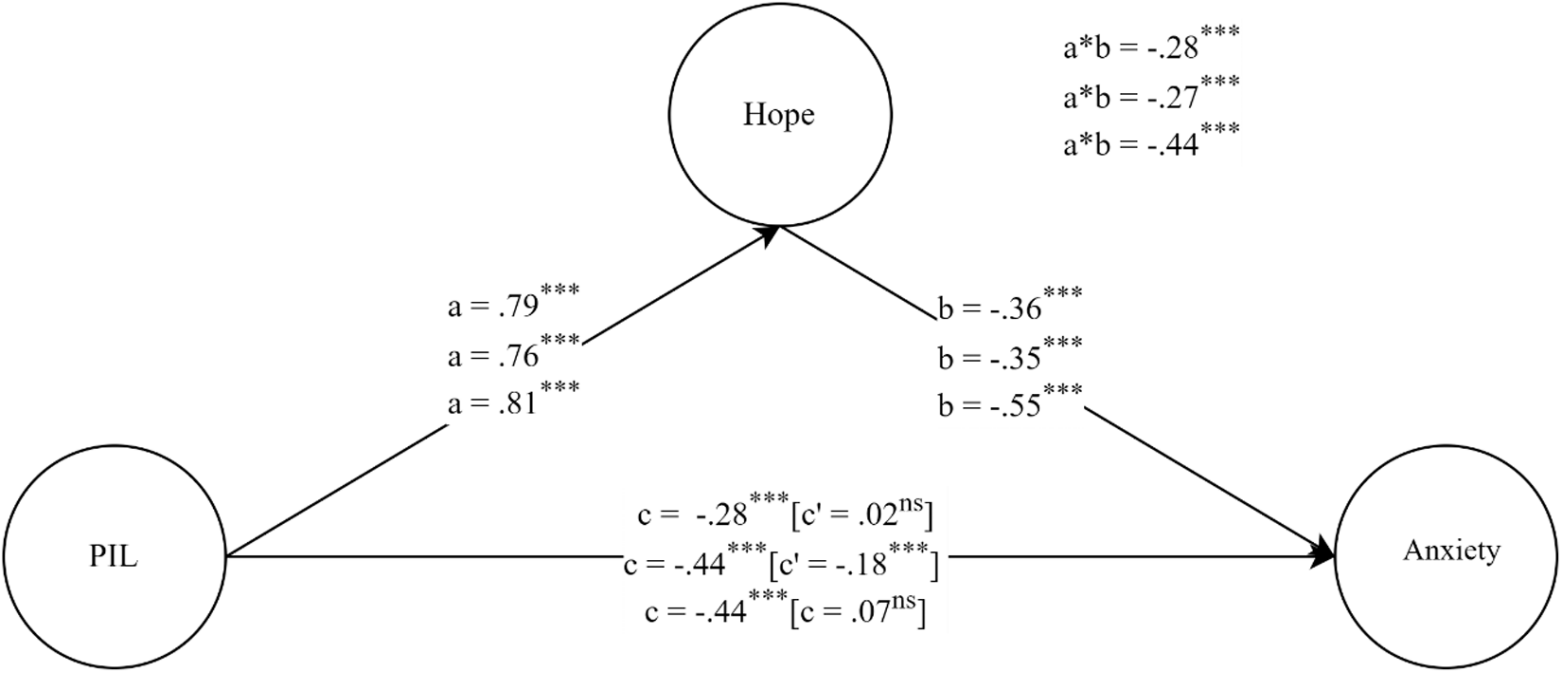
Mediation model of hope in the relationship between purpose in life and anxiety. χ2(831)= 2622.77; p < .05; CFI = .99; TLI = .99; RMSEA = .067 IC90% [.064 −.070]; SRMR = .074. ns = non-significant; *** p < .001. Error terms and item indicators were removed for clarity.

Fig 2 presents the mediation model results using SEM, incorporating the mediating role of hope. The direct effect (c’) of PIL significantly decreased and became non-significant in Bolivia and Ecuador (p > .05), indicating full mediation by hope in these countries. In contrast, in Colombia, the direct effect (c’) of PIL remained significant (p < .05), suggesting partial mediation.

Overall, PIL emerged as a strong positive predictor of hope, with a high and consistent effect across all three countries (β = 0.79, p < .001). In turn, hope functioned as a moderate negative predictor of anxiety, with similar values in Bolivia and Colombia but a stronger effect in Ecuador. Consequently, the indirect effect of PIL on anxiety, mediated by hope, was moderately negative in Ecuador and slightly negative in Bolivia and Colombia. The total effect (c) of PIL on anxiety was negative and of low-to-moderate magnitude in Bolivia, while higher in Colombia and Ecuador. Thus, in these countries, higher levels of PIL were associated with lower levels of anxiety.

Furthermore, regarding structural regression, the results indicated that PIL and hope, as exogenous variables, explained 24.4% of the variance in anxiety in Ecuador and 24.3% in Colombia, while in Bolivia, this percentage was lower, at only 11.7%. At a global level, the model explained 20.1% of the variance in anxiety, supporting the role of PIL and hope in anxiety.

### Cross-national equivalence of the mediation model

For the multigroup analysis (MG-CFA), the proposed mediation model was initially evaluated without constraints, as shown in Table 5. This model exhibited a good fit, confirming the basic equivalence of the model’s factorial structure. Subsequently, progressive constraints were imposed on factor loadings, thresholds, and residuals. The differences in ΔCFI and ΔRMSEA confirmed that the mediation model achieved strict invariance. This indicates that the configuration of latent variables, item factor loadings, thresholds, and residuals are equivalent across the three countries.

**Table 5:** Measurement equivalence between countries in the mediation model.

*Constrains*	*χ*^*2*^ *(df)*	*CFI*	*RMSEA*	*Δ χ*^*2*^ *(df)*	*p*	*ΔCFI*	*ΔRMSEA*
Configural	2429.0 (681)	.986	.073	–	–	–	–
Metric	2484.9 (721)	.986	.071	55.89 (40)	.049	.000	.002
Scalar	2622.8 (831)	.985	.067	137.84 (110)	.037	.000	.004
Strict	2772.6 (877)	.979	.067	149.80 (46)	.000	.001	.000

*** = **p* *< .001; * = p < .05; *+ = p > *.05; *χ*^*2*^* *=* *chi-squared; df = degrees of freedom; χ^2^/gl = normed chi-squared; CFI = Comparative fit index; TLI = Tucker Lewis Index; RMSEA = Root mean square error of approximation; SRMR = Standardized Root Mean Square Error Residual.

However, additional analyses are needed to explore potential differences in the model’s direct and indirect effects, as they may provide valuable insights into cultural influences on the relationship between PIL, hope, and anxiety.

### Mediation model adjusting direct and indirect effects

Based on the previous findings, an MG-SEM analysis was conducted to identify potential cultural differences in the direct and indirect effects among the variables. A total of three constrained models were analysed, in addition to Model 1, which included no constraints (Table 6).

**Table 6:** Models of equivalence of direct and indirect effects across countries.

Model	*χ* ^*2*^	*df*	Δ *χ*^*2*^	Δ *χ*^*2*^ *p*	CFI	TLI	SRMR	RMSEA
Model 1	2429.05***	681	–	–	.99	.98	.074	.073
Model 2	2448.22***	683	19.17	.000	.99	.98	.074	.073
**Model 3**	2432.02***	**683**	**2.97**	**.227**	**.99**	**.98**	**.074**	**.073**
Model 4	2448.33***	683	19.29	.000	.99	.98	.074	.073

Model 1: unconstrained, Model 2: constraint GAD-7 ~ PIL, Model 3: constraint HHI ~ PIL, Model 4: constraint GAD-7 ~ HHI.

Comparison with the baseline model, based on the fit indices (CFI, TLI, SRMR, and RMSEA) along with the chi-square difference test (Δχ^2^), indicated that Model 3, which constrained the effect of PIL on hope, demonstrated the best fit. These results suggest that the effect of PIL on hope is equivalent across countries (Bolivia: *β* = 0.79, *p* < .001; Colombia: *β* = 0.76, *p* < .001; Ecuador: *β* = 0.81, *p* < .001), while significant differences exist in the specific effects of PIL and hope on anxiety.

## Discussion

The objectives of this study included analysing the latent covariance between PIL, hope, and anxiety, assessing the mediating effect of hope in the relationship between PIL and anxiety, and evaluating the cross-national measurement equivalence of the model in university students from Ecuador, Colombia, and Bolivia.

The results indicate a high presence of PIL and a moderate level of hope among participants, with homogeneous values across countries. University life appears to influence purpose and meaning in life, with hope serving as a key factor in personal development [18,19,30]. Additionally, mild levels of anxiety were observed, reflecting mental health concerns within university populations [4–6].

SEM analyses revealed moderate negative correlations between PIL and anxiety, aligning with previous studies in the general population [25,26]. Negative correlations were also observed between hope and anxiety consistent with findings in both the general population [35,36] and university students [37,38]. Additionally, PIL and hope demonstrated a strong positive covariance [41]. These findings support the structural configuration of a well-fitted model.

Furthermore, the mediation model shows that PIL and hope jointly predict 20.1% of the variance in anxiety. In Colombia, PIL exerts both direct and indirect effects on anxiety through hope, whereas in Ecuador and Bolivia, only full mediation is observed. Hope acts as a key mediator in the association between PIL and anxiety, highlighting its organisational potential in psychological interventions [43]. This structural model represents an advancement in understanding anxiety based on positive mental health predictors.

Although the mediation model was invariant across the three countries—indicating that the general structure of relationships among variables is comparable—the direct and indirect effects differed. The only equivalent effect among the countries was that of PIL on hope, highlighting its contribution to the development of meaning, expectations, and vision for the future. Although these are preliminary results, the stronger association between hope and anxiety in Ecuador stands out, suggesting that in this cultural context, hope may play a more significant role as a protective variable. In contrast, the total effect and R-squared value in Bolivia were lower than in the other countries, indicating that although the variables were negatively related to anxiety, they do not carry the same weight in that context. These cross-national differences represent preliminary findings that can be further explored in future studies, considering the role of hope and PIL in accordance with each context and reality. Despite these variations, the results suggest that these positive psychological variables are associated with lower levels of anxiety across the three countries (see [26,40]).

These findings contribute to the study of anxiety in South American university students, offering a perspective grounded in protective mental health factors. Additionally, they propose innovative explanatory models regarding the impact of PIL and hope on anxiety. From a practical perspective, the study highlights the potential of interventions focused on strengthening these positive attributes, considering them as effective coping strategies for managing anxiety [12,13].

### Limitations

Although the current study proposes predictive elements and data projections, it does not establish causality between these positive predictors and anxiety. While the results theoretically suggest such a relationship, causality cannot be demonstrated due to the constraints of cross-sectional and non-experimental studies. Moreover, the sample used was not clinical, consisting mainly of students with low to moderate anxiety levels. This could have attenuated the observed correlations. These associations could differ between individuals with clinical levels of anxiety. Future research should address this limitation by employing longitudinal methodologies with at least three measurement points or experimental designs to better assess causal effects, and by including clinical samples to deepen understanding of the relationships found.
